# Regulation of divergent cortisol responsiveness in European sea bass, *Dicentrarchus labrax* L.

**DOI:** 10.1371/journal.pone.0202195

**Published:** 2018-08-10

**Authors:** Athanasios Samaras, Michail Pavlidis

**Affiliations:** Department of Biology, University of Crete, Heraklion, Crete, Greece; National Cheng Kung University, TAIWAN

## Abstract

Mechanisms regulating differences in cortisol responsiveness between low (LR) and high response (HR) individuals have been poorly studied. In this context, we aimed to study key regulatory processes in cortisol dynamics at the head kidneys of LR and HR European sea bass. To do so, resting plasma cortisol and ACTH concentrations were quantified in these fish. Additionally, the head kidneys of these individuals were superfused through an *in vitro* superfusion system and stimulated with the same amount of ACTH to assess their cortisol biosynthetic capacity. Moreover, the expression of important genes in cortisol regulation was assessed. Results showed that LR fish had lower resting cortisol concentrations than HR, although no differences existed in the circulating levels of ACTH. Additionally, the biosynthetic capacity of HR was higher than that of LR fish when *in vitro* stimulated with ACTH. At the molecular level, a statistically significant 3.4-fold higher expression of the ACTH receptor, *mc2r*, and a 2.3-fold, though not significant, higher expression of *11β-hydroxylase* (*cyp11b1*), an enzyme involved in cortisol biosynthesis, was observed in the HR fish. Finally, a statistically significant 1.3-fold lower expression of *11β-hydroxysteroid dehydrogenase 2* (*hsd11b2*), an enzyme involved in cortisol inactivation, was observed in HR when compared to LR fish. Therefore, it was for the first time indicated that cortisol dynamics can also be regulated at the post-production level in the head kidney. Collectively, our results highlight the crucial role of the interrenal tissue in the regulation of differences in cortisol response between LR and HR sea bass individuals.

## Introduction

Cortisol is considered the major stress hormone in marine teleost fish, regulating many metabolic actions for the redistribution of energy towards survival mechanisms and away from growth and reproduction [1–2]. In mammals, cortisol is synthetized at the adrenal gland. Fish, however, do not have a discrete adrenal gland, and cortisol is produced at the interrenal cells, which are distributed in the head kidney [3]. Cortisol production, secretion and actions are under complex control, regulated by the Hypothalamus–Pituitary–Interrenal (HPI) axis (see [3–4] for review).

Briefly, the *nucleus preopticus* (NPO) at the hypothalamus produces corticotropin-releasing hormone (CRH) that stimulates the pituitary *pars distalis* to produce the adrenocorticotropic hormone, ACTH. This hormone subsequently stimulates the interrenal cells at the head kidney to produce cortisol [5–6]. ACTH acts by binding to an ACTH-specific receptor, namely the melanocortin 2 receptor (MC2R) [7–9]. The expression of the *mc2r* gene is crucial for the regulation of cortisol biosynthesis from the interrenal cells [9]. Once ACTH binds to MC2R at the interrenal tissue, a series of enzymatic reactions takes place to produce cortisol. The final step of this enzymatic reactions is the conversion of 11-deoxycortisol to cortisol, catalyzed by 11b-hydroxylase (CYP11B1) [10]. Moreover, cortisol can be inactivated to cortisone by 11b-hydroxysteroid dehydrogenase 2 (HSD11B2) [10–11]. Finally, cortisol is released into the circulation, where it stimulates various target-tissues regulating their actions. These actions are mediated by cortisol receptors, which in most teleost species are two glucocorticoid (GRs) and one mineralocorticoid receptors (MR) [3; 12].

Divergence in cortisol responsiveness between individuals of the same species has been long described in fish [13–16] and other vertebrates [17–18]. Subsequently, animals that consistently show low (LR) or high (HR) cortisol circulating levels after exposure to stressors have been characterized [13–16]. These phenotypes have been associated with differences in performance, such as growth [19–21], and hepatic metabolic processes [16,22], as well as behavioral differences [23–24]. Still, however, the underlying mechanisms regulating this individual cortisol responsiveness have not been extensively described.

The role of the hypothalamus in the regulation of individual responses is still ambiguous. Specifically, no differences were found in resting *crh* mRNA expression between LR and HR individuals of rainbow trout, *Oncorhynchus mykiss* [25]. Additionally, the concentration of the pituitary-derived ACTH was also not significantly different between unstressed or stressed LR and HR trout [26].

On the contrary, the interrenal tissue can play a crucial role in the regulation of cortisol responsiveness between LR and HR fish [26]. In rainbow trout, higher cortisol production in HR compared to LR fish has been observed after stimulation with ACTH [26]. Moreover, higher expression of *mc2r* in the head kidney of these fish has been reported [27]. Additionally, an upregulation of mRNA expression of genes encoding for enzymes regulating cortisol synthesis (StAR, P450scc, 3βHSD) has been observed in HR individuals of Atlantic cod, *Gadus morhua*, [15].

Recently, individual divergent cortisol responsiveness has been described in European sea bass, and LR and HR individuals have been characterized [16]. These fish, apart from differences in their post-stress plasma cortisol concentrations, have also shown differences in the resting and free post-stress cortisol levels, as well as the hepatic transcription profile [16]. In this context, the aim of the present study was to estimate the *in vitro* cortisol production capacity of LR and HR sea bass, as well as study the mRNA expression of significant for cortisol regulation genes, *i*.*e*. *mc2r*, *cyp11b1*, and *hsd11b2*, in the head kidney of these fish.

## Materials and methods

### Fish and husbandry conditions

Sea bass previously identified as LR and HR individuals as described by [16] were used. Fish originated from the Nireus S.A. (Greece) family-based breeding program. Briefly, 960 individually PIT-tagged fish belonging to 96 families were analysed for cortisol concentrations after exposure to acute chasing stress, once per month and for three consecutive months. The stress protocol consisted of lowering the water of the tank and chasing fish with a net for 5 minutes. 30 min post-stress fish were anesthetized in ethylene glycol monophenyl ether (300ppm; Merch 807291; USA) and sampled. Cortisol levels were normalized using the z-score normalization and their cumulative z-score of the three-successive sampling were ranked. Those fish showing the highest cumulative z-scores (highest 10% of the distribution) were characterized as HR, and those showing the lowest scores (lowest 10%) as LR fish. These fish were placed together in a 2.5 m^3^ circulating tank at Nireus S.A. research facilities (Pyrgoulaki, Euboea, Greece).

Fish were fed twice per day, for 6 days a week, using a commercial diet (Blue Line 45:20 3.5 mm, Feedus S.A., Greece). During the experiment fish were monitored by the designated veterinarian, while water quality was checked daily. Photoperiod was set at 12L:12D, water temperature was 18.20 ± 0.03°C, and salinity 27. Oxygen and pH ranged between 6–10 mg L^-1^ and 7.20–7.40, respectively. The experiment was carried out at Nireus S.A. research facilities.

### Sampling

In order to perform the superfusion experiment, fish were randomly sampled from the tank by the use of a baited-hook. This was performed to immediately capture fish avoiding stress and minimizing disturbance to the remaining fish which were to be sampled during the next days of the experiment. No bias in terms of number of fish that bit the bait was observed, since in total the same number of LR and HR fish were caught, while in each sampling day both LR and HR fish were sampled.

At the time of sampling fish were 34-months old, weighting 581.9 ± 175.9 g (mean ± SD). Each day of the experiment 4 fish were sampled, and immediately euthanatized in high dose of 2-phenoxyethanol (500 ppm; Merck 80729, USA). In total 6 LR (3 females / 3 males) and 6 HR (4 females / 2 males) fish were used in this experiment. Blood was immediately drawn from the caudal vein via heparinized syringes, and fish were dissected to collect the head kidneys, which were placed in the superfusion medium. Specifically, both head kidneys were dissected from each fish, minced into fragments of approximately ~1 mm^3^, and mixed in order to avoid possible differences between the left and right head kidneys. Part of the tissue was immediately frozen in dry ice and stored at -80°C until the analysis. The rest of the head kidney was placed in the superfusion medium to remove the excess of blood. The medium was refreshed 4 times, until no signs of blood were observed. Afterwards, approximately 200 mg of tissue from each fish were placed in the superfusion chambers.

### Superfusion

Head kidneys were superfused with carbogen saturated (95% O_2_/5% CO_2_) 15 mM HEPES/Tris buffer (pH 7.4) containing 171 mM NaCl, 2 mM KCl, 2 mM CaCl_2_, 0.25% (w/v) glucose, 0.03% (w/v) bovine serum albumin and 0.1 mM ascorbic acid. The medium was delivered to the superfusion chambers at a constant rate of 30 μl min^-1^ via a peristaltic pump (Minipuls 3; Gilson Inc., WI, USA). Superfused medium dribbled from the chamber at the same rate and was collected for cortisol analysis. Samples were collected every 30 min, before the hormone treatment, and over 5- or 15-min intervals after the hormone addition and stored at -20°C until analysis. After 150 min of superfusion, when the release rate has reached a baseline release (as seen by the superfusion of the tissue without ACTH treatment; data not shown), the head kidneys were superfused with 10^−7^ M of human ACTH_1-39_ (Sigma-Aldrich^®^ A042, USA) dissolved in the superfusion medium. The concentration was chosen according to a previous study that showed efficient stimulation of cortisol production by hACTH in sea bass at doses between 10^−9^ to 10^−7^ M, with an estimated EC_50_ at 0.49 x 10^−8^ M hACTH [28]. However, since the superfusion protocol used by [28] delivered approximately 2 times higher amount of the medium containing ACTH compared to our protocol (due to a higher superfusion rate), we chose to use a higher than the EC_50_ dose (*i*.*e*. 10^−7^ M), similar to that of [7] in rainbow trout. This dose efficiently stimulates cortisol production in sea bass, without any pharmacological effects [28].

### Analytical procedures

Plasma cortisol and cortisol released in the superfusion medium by the head kidney were measured using a commercial enzyme-linked immunoassay kit (DRG^®^ Cortisol ELISA, DRG^®^ International Inc, Germany), previously evaluated in this species [29]. Plasma ACTH concentrations were assayed by a commercial, specialized for fish, enzyme-linked immunoassay kit (CUSABIO^®^ Fish Adrenocorticotropic hormone ELISA kit, CUSABIO BIOTECH Co. Ltd, China). This assay is based on competitive inhibition reaction between the ACTH of the samples and externally added biotin-conjugated ACTH, with the pre-coated antibody specific for ACTH. No significant cross-reactivity or interference between fish ACTH and its analogue, α-MSH, was observed when we tested the performance of the kit against sequential dilutions of human α-MSH (Sigma-Aldrich^®^, M4135, USA). Both intra- and inter-assay variation were less the 15% (CV% < 15). The standard curve and the extrapolation of the results were analyzed using the Curve Expert 1.3 software (CurveExpert, USA), according to manufacturer’s instructions, using a four-parameter logistic curve-fit.

### RNA purification and cDNA synthesis

Head kidney samples (20–30 mg) were disrupted and homogenized using the TissueRuptor (Qiagen, Hilden, Germany) for 20 s in 350 μl LBP lysis buffer (NucleoSpin^®^ RNA Plus, MACHEREY-NAGEL GmbH & Co. KG, Düren, Germany). Total RNA was isolated using the NucleoSpin^®^ RNA Plus kit (MACHEREY-NAGEL GmbH & Co. KG, Düren, Germany), according to manufacturers’ instructions. RNA yield and purity were determined by measuring the absorbance at 260 and 280 nm, using Nanodrop^®^ ND-1000 UV-Vis spectrophotometer (Peqlab, Erlangen, Germany), while its integrity was tested by 1% agarose gel electrophoresis. Reverse transcription (RT) was performed using 1 μg RNA with the QuantiTect Reverse transcription kit (Qiagen, Valencia, USA), following manufacturer’s instructions. One LR and one HR samples were excluded from the analysis due to insufficient RNA yield and purity.

### Quantitative real-time PCR (qPCR)

The mRNA expression of genes encoding for *mc2r*, *hsd11b2*, and *cyp11b1* was determined with quantitative polymerase chain reaction (qPCR) assays using the KAPA SYBR^®^ FAST qPCR kit (Kapa Biosystems, Wilmington, USA). Oligonucleotides used in the qPCR analysis are shown in Table 1 ([30]; Tsalafouta, unpublished data). Reactions were cycled, and the resulting fluorescence was detected with MJ Mini Thermal Cycler (Bio-Rad) under the following parameters: (1) 95°C for 3 min (HotStarTaq DNA Polymerase activation step), (2) 94°C for 15 s (denaturation step), (3) 60°C for 30 s (annealing step), (4) 72°C for 20 s (extension step), cycling steps (2) to (4) for 40 cycles. Levels of *mc2r*, *hsd11b2*, and *cyp11b1* mRNA were normalized based on the reference genes *actin beta 1* (*actb1*), *eukaryotic translation elongation factor 1A* (*eef1a*), and *18s*. A relative standard curve was constructed for each gene, using 4 sequential dilutions (1:5) of a pool of all the cDNA samples. geNORM analysis was also performed to validate which were the most suitable reference genes to serve as internal control and showed that *18s*, and *eef1a* were the most appropriate.

**Table 1:** Primer sequences used in qPCR for sea bass.

Gene	Forward Primer 5’ to 3’	Reverse Primer 5’ to 3’
*actb1*	CGCGACCTCACAGACTACCT	AACCTCTCATTGCCGATG
*eef1a*	GCCAGATCAACGCAGGTTACG	GAAGCGACCGAGGGGAGG
*18s*	TCAAGAACGAAAGTCGGAGG	GGACATCTAAGGGCATCACA
*mc2r*	CATCTACGCCTTCCGCATTG	ATGAGCACCGCCTCCATT
*hsd11b2*	CACCCAGCCACAGCAGGT	ACCAAGCCCCACAGACC
*cyp11b1*	GGAGGAGGATTGCTGAGAACG	AGAGGACGACACGCTGAGA

### Statistical analysis

Statistical analysis was performed using the SPSS v22.0 (IBM Statistics for Windows; IBM Corp., Armonk, NY, USA), while the figures were created using GraphPad Prism 6.0 (GraphPad Software Inc., La Jolla, CA, USA). Prior to analysis, data were tested for normality using the Kolmogorov-Smirnov & the Shapiro-Wilk tests. Plasma cortisol, ACTH and head kidney gene expression data of LR and HR fish were analysed using t-tests. In the case of *mc2r* and *cyp11b1* normality criteria were not met, and the non-parametric Mann-Whitney test was used. Correlations between plasma and gene expression variables were performed using the Spearman Rank test.

In the superfusion experiment, a two-way repeated measures Analysis of Variance (ANOVA) was performed on cortisol release rate, using a mixed design with one between factor (*type of response*, *i*.*e*. LR or HR) and one within factor (*superfusion time*), which consisted the repeated measured factor. Data were tested for sphericity using Mauchly’s test, while the most stringent Greenhouse-Geisser correction F-test was used to determine significant effects of *type of response*, *superfusion time*, and their interaction. When significant differences existed, Tukey’s post-hoc tests were used. All tests were performed with *P* < 0.05 set as the levels of significance.

To estimate the magnitude of cortisol response after superfusion of the head kidneys with ACTH, the Area Under the Curve (AUC) was calculated based on the trapezoidal method, using the GraphPad Prism 6.0 (GraphPad Software, USA). Moreover, since each sample had a different baseline cortisol release rate (t = 150 min), the AUC with respect to each specific baseline value was calculated as described by [31]. Differences between the mean AUC of LR against HR fish were tested using the non-parametric Mann-Whitney t-test, since the criteria for a parametric test were violated.

All the above described analyses were also performed to check possibly differences between male and female fish. No differences were observed between sexes, and therefore data are not presented on the results.

### Ethical statement

The stress protocol applied on fish for the initial characterization of LR and HR individuals consisted of lowering the water of the tank and chasing fish with a net for 5 minutes. Subsequently, fish were anesthetized in ethylene glycol monophenyl ether (300ppm) and sampled 30 min post-stress. Fish during the experiment were monitored by the designated veterinarian, while water quality was checked daily. The selected fish used for molecular analysis were euthanized immediately after capture in high dose of anaesthesia. Nireus S.A. research facilities are certified and have obtained the codes for the rearing and use of fish for scientific purposes (EL04-BIOexp-01). All procedures on fish used at this study were approved by the Research Ethics Committee of the Department of Biology, University of Crete, following the Three Rs principle, in accordance with Greek (PD 56/2013) and EU (Directive 63/2010) legislation on the care and use of experimental animals.

## Results

### Plasma cortisol and ACTH

**Fig 1:**
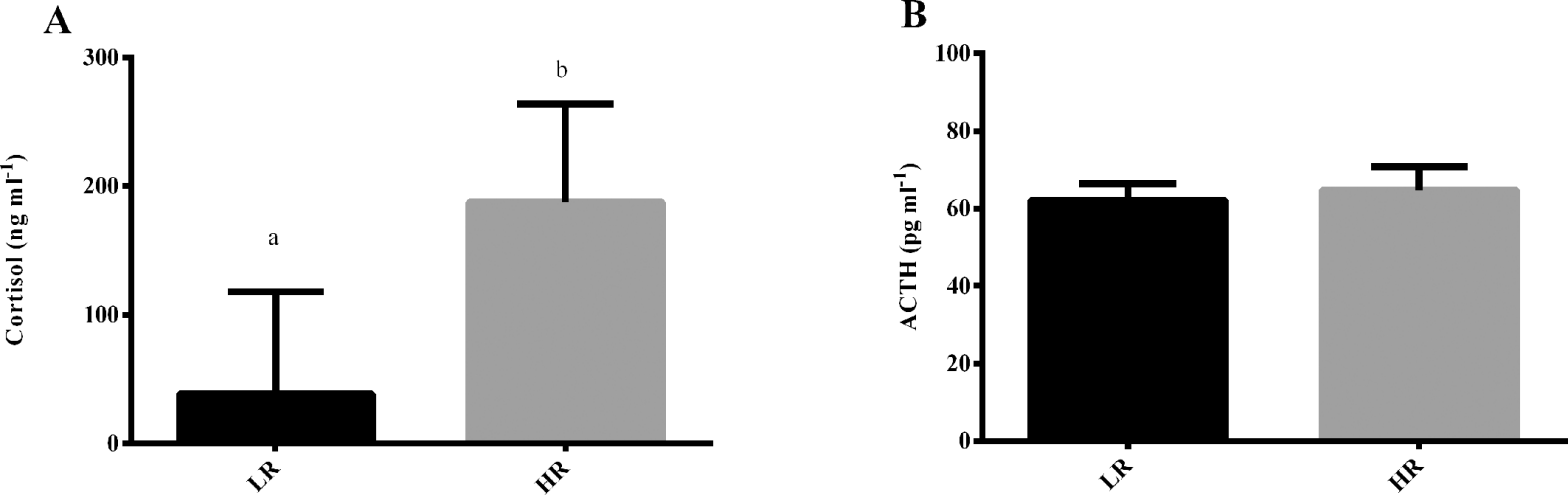
Resting plasma cortisol and ACTH concentrations of LR and HR sea bass. Cortisol (A) and ACTH (B) concentrations are presented as means + SD (*n* = 6). Means with different letters differ significantly from one another (t-test; *P* < 0.05).

Resting levels of plasma cortisol were significantly higher in HR than LR fish (t_10_ = 3.328; *P* = 0.008) (Fig 1A). Plasma ACTH concentrations, on the other hand, showed no statistically significant differences between the two groups (t_10_ = 0.879; *P* = 0.400) (Fig 1B).

### Superfusion

**Fig 2:**
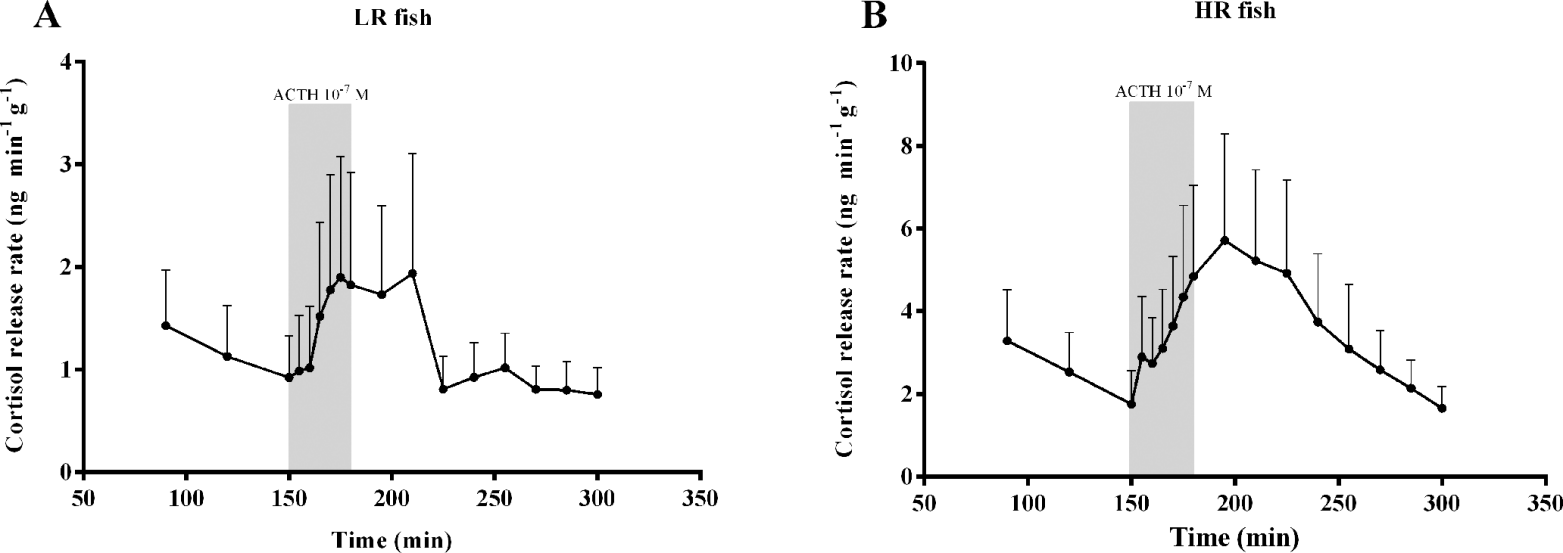
*In-vitro* release of cortisol from superfused head kidneys of LR (A) and HR (B) sea bass stimulated with ACTH. Values are expressed as cortisol release rate (ng min^-1^ g^-1^) and presented as means + standard error of the mean (*n* = 6). The y-axis is in different scale. The shaded areas represent the period over which the head kidneys were treated with 10^−7^ M ACTH.

Cortisol release rate, expressed as the amount of cortisol released (ng) in respect to time (min) and tissue weight (g), showed an increase after ACTH stimulation in both LR and HR fish (Fig 2). No statistically significant differences existed between the response curves of LR and HR fish (F_1,10_ = 2.034; *P* = 0.184). However, when the mean Areas Under the Curve (AUC) of the response (*i*.*e*. after the administration of ACTH) were compared, a higher overall response was observed in the HR fish (mean ± SD: 182.71 ± 212.82 ng g^-1^ in LR and 559.78 ± 560.56 ng g^-1^ in HR fish, respectively) (Τ = 4.000; *P* = 0.026).

**Fig 3:**
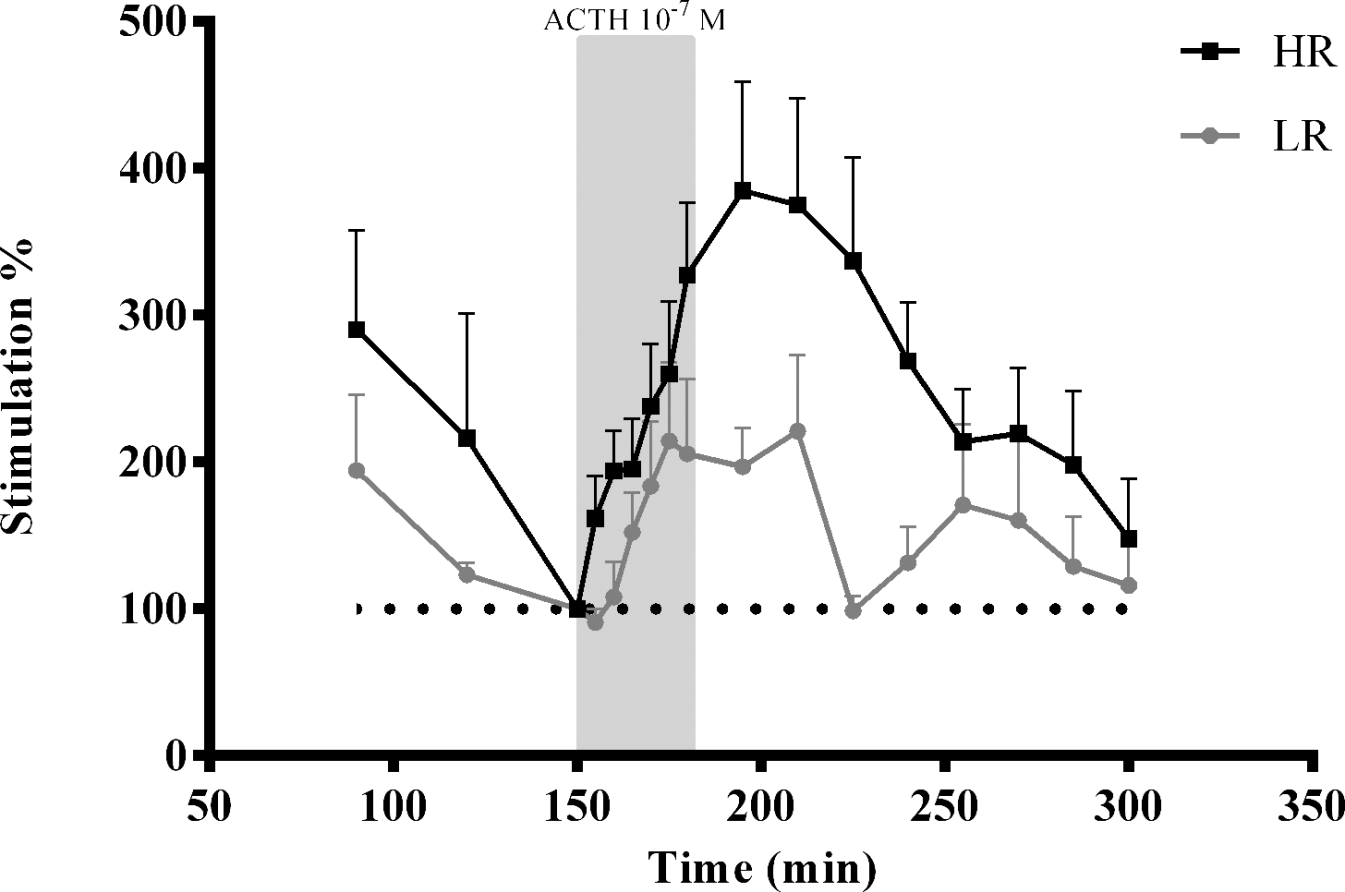
*In-vitro* percental release of cortisol from superfused head kidneys of LR and HR sea bass individuals stimulated with ACTH. Values are expressed as percental secretion relative to basal and presented as means + SD (*n* = 6). The shaded areas represent the period over which the head kidneys were treated with 10^−7^ M ACTH.

When the relative stimulation of cortisol secretion was analysed, calculated by setting cortisol release prior to ACTH administration (*i*.*e*. 150 min) to 100%, a significant stimulation was observed in both LR and HR fish, as shown by the Greenhouse-Geisser F-test (F_3.27,32.70_ = 5.171; *P* = 0.004) (Fig 3). Moreover, the release in HR fish was significantly higher than in LR fish (F_1,10_ = 5.154; *P* = 0.047) (Fig 3).

### Gene expression

**Fig 4:**
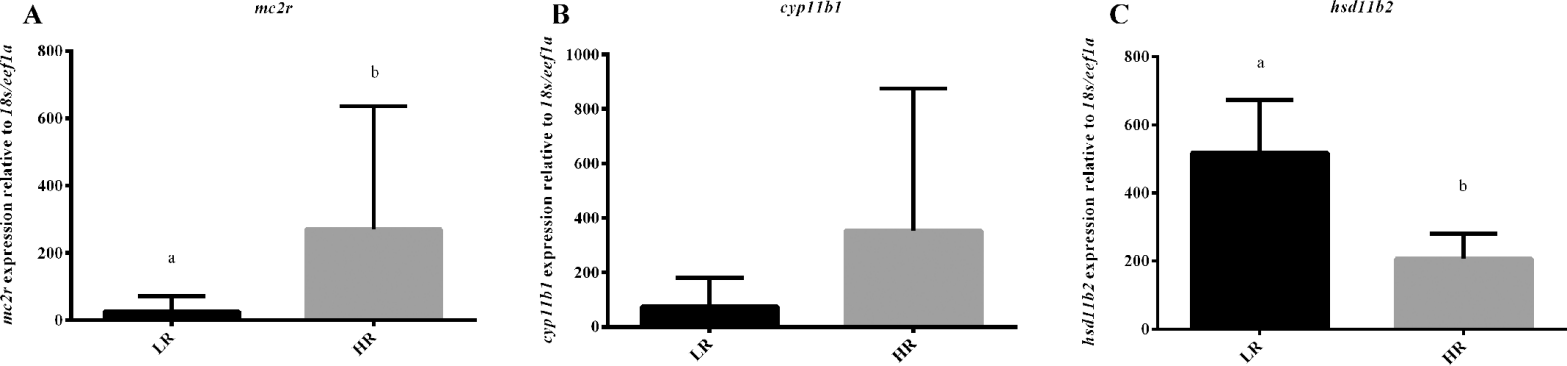
Relative mRNA transcript levels of *mc2r* (A), *cyp11b1* (B), and *hsd11b2* (C) in the head kidneys of LR and HR sea bass. Values are presented as means + SD (*n* = 5). Means with different letters differ significantly from one another as shown by t-tests (t-tests; *P* < 0.05).

Analysis of the relative mRNA gene expression showed that LR and HR fish differed in the expression of *mc2r* and *hsd11b2*. Specifically, *mc2r* was significantly more expressed in HR compared to LR fish (T = 2.000; *P* = 0.032) (Fig 4A). The opposite pattern was observed for *hsd11b2*, being significantly more expressed in LR than HR fish (t_8_ = 4.027; *P* = 0.004) (Fig 4C). Finally, a 2.3-fold, though not statistically significant (T = 6.000; *P* = 0.222), higher expression of *cyp11b1* was observed in HR compared to LR (Fig 4B).

### Correlations

The Spearman rank correlation coefficients between plasma concentrations of cortisol and ACTH, as well as the expression of genes and the cortisol superfusion release rate were calculated (Table 2). Significant correlations existed between plasma cortisol, the expression of *mc2r* and the maximum superfusion release rate. A significant correlation between the expression of *mc2r* and *cyp11b1* was also observed. Finally, the expression of *cyp11b1* was significantly correlated with the basal and maximum superfusion cortisol release rates.

**Table 2:** Spearman rank correlation coefficient, *r*, between the concentration of plasma cortisol and ACTH, the relative gene expression of *mc2r*, *cyp11b1*, and *hsd11b2* and cortisol superfusion release rates (*n* = 6 for plasma; *n* = 5 for mRNA).

	ACTH	*mc2r*	*cyp11b1*	*hsd11b2*	F basal	F max
Cortisol	0.573	0.697*	0.588	-0.248	0.327	0.692*
ACTH		0.527	0.442	-0.127	0.313	0.357
*mc2r*			0.721*	-0.491	0.500	0.818**
*cyp11b1*				-0.212	0.701*	0.697*
*hsd11b2*					-0.037	-0.455
F basal						0.756**

Cortisol superfusion release rates are indicated as F basal, for the basal (*i*.*e*. prior to ACTH stimulation) and F max for the maximum post-ACTH stimulation cortisol release rate. Significant correlations are marked with asterisks (* *P* < 0.05; ** *P* < 0.01).

## Discussion

Results of the present study demonstrate that differences in resting circulating cortisol levels can be observed between LR and HR sea bass characterized based on their stress response. On the other hand, no differences existed in resting plasma ACTH concentrations. Moreover, a differential expression of important genes in the regulation of interrenal sensitivity to ACTH and cortisol metabolism was observed between LR and HR individuals. Finally, *in vitro* stimulation of the head kidneys of LR and HR fish with the same concentration of hACTH showed that HR fish had a greater overall outcome and higher relative-to-basal cortisol production compared to LR. It should be noted that the use of the commercially available hACTH should not impede the results of the present study since the structure of ACTH, especially the HFRW and RKRRP (KKRRP in human) core sequences required for receptor binding, are highly conserved, specifically shared from fish to mammals [7,32–34]. The substitution of arginine in teleosts to lysine in human in the R(or K)RRP motif has not been shown to interfere with the activation of non-mammalian MC2R by hACTH [32]. This is further supported by the fact that hACTH has been shown to stimulate the ACTH receptor MC2R in sea bass [9].

The above-mentioned results suggest that ACTH concentration is not the factor regulating cortisol differences between LR and HR fish. Specifically, no differences were observed in plasma ACTH concentrations between these phenotypes, while HR fish showed a higher *in vitro* ACTH-stimulated cortisol production from the head kidneys. No direct estimates on the regulation of the brain compartments of the HPI axis (*i*.*e*. hypothalamus and pituitary) were made in the present study. Instead, plasma ACTH was measured as an indicator of the outcome of the Hypothalamus–Pituitary axis, since ACTH is the pituitary’s endpoint product, as far as the HPI is concerned, given the yet unclear role of α-MSH in the stress response [35]. In fact, literature regarding the regulation of divergent cortisol responsiveness by the hypothalamus and pituitary is still scarce in fish. No differences in the mRNA expression of CRH between LR and HR trout have been reported, although post-stress CRH expression seems to be higher in HR than LR trout [25]. Moreover, although external administration of CRH has led to divergent cortisol responses in LR and HR male striped bass, *Morone saxatilis*, it is not clear whether CRH was the driving force regulating these differences [14]. Additionally, in agreement with the present study, no differences in resting and post-stress ACTH circulating levels existed between LR and HR phenotypes in rainbow trout [26].

On the other hand, the present study revealed that the interrenal tissue plays a major role on the regulation of divergent cortisol responsiveness. Specifically, when head kidneys of LR and HR were *in vitro* stimulated by ACTH the overall production of cortisol was higher in the latter. These results highlight the importance of the interrenal tissue in the regulation of individual cortisol responsiveness. Similar results have been observed in rainbow trout, where HR fish produced higher cortisol amounts than LR when *in vivo* stimulated with ACTH [26]. Additionally, differences in the adrenocortical capacity and sensitivity to ACTH between strains with divergent cortisol responsiveness have also been described in other vertebrate taxa, such as birds [36–37], and mammals [38–39].

The head kidney mRNA expression analysis of genes involved in the regulation of biosynthesis (*cyp11b1*), and degradation (*hsd11b2*) of cortisol, as well as that of the receptor of ACTH (*mc2r*) were examined in order to get a better insight in the mechanisms regulating differential cortisol dynamics between LR and HR fish. Results pointed out that divergent cortisol responsiveness could in part be regulated by differences in the sensitivity of the interrenal tissue to ACTH and processes in cortisol metabolism. It should, however, be kept in mind that these data refer to gene expression and care should be taken since no data for the actual protein levels were analysed.

In details, the sensitivity of the tissue to ACTH, as depicted by the expression of its receptor, *mc2r*, was higher in HR than LR fish. Moreover, the significant correlation between the expression of *mc2r* and resting plasma cortisol levels suggests that the expression of *mc2r* between LR and HR fish could partly explain the differences in resting cortisol concentrations between these phenotypes. A higher expression of *mc2r* in HR than LR fish has also been reported in rainbow trout head kidney [27], leading these authors to suggest that this explained the increased cortisol response to ACTH observed in HR fish by [26].

Although HR fish had higher plasma cortisol levels than LR fish, and despite that they responded more intensely when *in vitro* stimulated with ACTH, no statistical differences were observed in the expression of *cyp11b1*. In other fish species, such as the Atlantic cod, a differential regulation of the gene expression of enzymes involved in cortisol biosynthesis has been observed between LR and HR fish [15]. Specifically, the genes of the enzymes StAR, P450scc, and 3βHSD were significantly upregulated in HR compared to LR fish. It seems therefore that there is differential regulation in the expression of genes involved in cortisol production between LR and HR fish, although this was not statistically supported for *cyp11b1* in the present study, despite the 2.3-fold difference, probably due to the high variability of the data.

In addition, the expression of *hsd11b2*, which encodes for the enzyme that inactivates cortisol to cortisone showed significantly reduced expression in HR compared to LR fish. To the author’s best knowledge, this is the first time that significant differences in the expression of this cortisol-catabolizing gene have been reported between LR and HR individuals in any vertebrate species. This enzyme can exert intracrine and endocrine effects. The former mainly concern organisms that produce aldosterone, such as mammals, where *hsd11b2* is essential in inactivating cortisol, a MR ligand with higher potency than aldosterone, in MR-rich tissues to avoid unnecessary MR activation [40–41]. In terms of endocrine effects, this enzyme regulates circulating levels of cortisol, and it has been suggested to inactivate approximately 30–40% of the total daily production [41]. Inhibition of *hsd11b2* has led to increased cortisol levels in zebrafish [42] and corticosterone in rats [43], reflecting its capacity to regulate the activity of the HPI axis.

Finally, our data on the molecular regulation of individual differences between LR and HR fish supplemented by the suggested genetic basis of this trait [16, 44] highlight its potential role as a selection criterion in selective breeding programmes. Especially, since stress is known to affect growth [20], as well as evoke reproductive dysfunctions and disease outbreaks [45], cortisol responsiveness could prove to be an important selection trait.

In conclusion, it seems that the differences in circulating cortisol levels between LR and HR sea bass are mainly regulated at the level of the interrenal tissue. Specifically, cortisol metabolism, mainly post-production, and interrenal sensitivity to ACTH seem to be crucial sites of regulation. When *in vitro* superfused with the same amount of hACTH, head kidneys of HR fish showed a higher cortisol biosynthetic capacity than LR. This higher cortisol production was accompanied by a combination of increased sensitivity of the interrenal tissue to ACTH, and a downregulation in the expression of a gene regulating degradation of cortisol in HR fish. These results can provide a better insight at the regulation of cortisol variability in sea bass and other teleost and vertebrate species.

## Supporting information

S1 Data Cortisol, ACTH and mRNA gene expression data. (XLSX)

S2 Data In vitro cortisol release rate expressed as ng min^-1^ g^-1^. (XLSX)
